# 3*dcon*: tomogram denoising by deconvolution

**DOI:** 10.1107/S205979832600999X

**Published:** 2026-10-12

**Authors:** Peter Kirchweger, Lev Melnikovsky, Shahar Seifer, Michael Elbaum

**Affiliations:** a Department of Chemical and Biological Physics, Weizmann Institute of Science, 7610001Rehovot, Israel; National Center of Biotechnology, CSIC, Spain

**Keywords:** cryo-ET, cryo-STET, 3D deconvolution, tomogram denoising, mitochondria

## Abstract

Cryo-electron tomography is an expanding technology for the study of macromolecules, viruses and cells. It is often applied to specimens that are too large or heterogeneous for methods based on 2D image averaging such as single-particle analysis, for example intracellular membranes or organelles. Current practice records a tilt series of projection images in rotation. Reconstruction is normally an ill-posed mathematical problem. Particularly for the under-determined case of sparse data, discrete tilt angles and a limited tilt range, characteristic artifacts appear in the reconstructed slices. Much of what appears as noise is in fact structural: the projection of contrast from different planes. Various schemes are employed to regularize the reconstruction, including machine-learning frameworks built on neural networks. To the extent that the noise is structural, based on the geometry of the tilt series, it might be suppressed by deconvolution with a suitable kernel. This was demonstrated and has been used regularly in cryo-STEM tomography of thick specimens where the under-sampling problem is particularly acute. Here, we present 3*dcon* as an open-source extension of the entropy-regularized deconvolution algorithm that had been adopted from fluorescence microscopy. It takes advantage of modern computing hardware for convenient and fast processing. Deconvolution is entirely algorithmic, meaning that successful processing of the data does not depend on the data itself. As such, it should be robust in a wide variety of applications.

## Abstract 2

An open-source entropy-regularized deconvolution workflow is presented for denoising tomographic reconstructions from cryo-electron microscopy.

## 1. Introduction

Tomography is a widely used technique in many fields of science and medicine. It may be defined rather generally as a reconstruction to higher dimension from projections, for example from two-dimensional (2D) images to a 3D volume. For the particular case of projections around a single axis of tilt, the Radon transform describes the transformation from radial to Cartesian coordinates. The inverse transform, from projections to volume, is the essence of tomographic reconstruction. In many cases this operation is ill-posed mathematically due to sparsity of the original data. Therefore, the reconstruction requires some form of regularization.

Cryo-electron tomography (cryo-ET; Frank, 2006 ▸), the case at hand here, is used to visualize unstained biological samples in near-native environments in 3D. For a given dataset size, there is a necessary compromise between resolution and field of view (FOV). In cryo-ET this begins with the microscope configuration. Typically, the wide-field modality of conventional transmission electron microscopy (TEM) is optimal for high resolution, while tomography based on scanning transmission electron microscopy (STEM) is better suited for larger FOV, and in particular for thicker specimens (Elbaum *et al.*, 2021 ▸; Varsano & Wolf, 2022 ▸).

In cryo-ET, the acquisition of a tomographic tilt series suffers from a number of characteristic limitations. Foremost, a planar specimen cannot be tilted perpendicular to the illumination, so there exists a large range of inaccessible tilt angles surrounding the surface normal; typically, the rotation is restricted to about −60° to 60°. This creates a large ‘missing wedge’ of information in the upward and downward orientations of the resulting 3D volume. Furthermore, the projections are normally recorded at discrete tilt angles due to the requirements of mechanical stability during the recording. The discrete tilt steps lead to smaller missing wedges in between the step directions (Penczek, 2010 ▸). More subtly, the sensitivity of delicate cryogenic specimens to the ionizing electron irradiation limits the total exposure rather strictly, so that a balance must be struck between the number of tilt views acquired and the dose applied to individual tilt images. The Crowther criterion equates roughly between the number of distinct acquired views and the number of independently determined virtual slices in the reconstruction (Crowther *et al.*, 1970 ▸). These geometric considerations introduce specific artifacts in the reconstruction, for example an elongation in *z*, anisotropic resolution and streaking artifacts (most notably on high-intensity features). In any given *xy* plane, much of what appears as random noise is actually a projection of intensity from distant planes. As a structural artifact, it should be possible to suppress this apparent noise based on knowledge of the reconstruction process.

The practical implementation of tomographic reconstruction involves two distinct steps. Alignment of the tilt series compensates for mechanical or optical deviations from the ideal of rigid-body rotation around a single axis, or even distortion of the sample itself that may occur during the tilt series (Fernandez & Li, 2021 ▸; Chen, 2026 ▸; Chaillet *et al.*, 2026 ▸). Reconstruction may then be performed by a number of related algorithms. These include most commonly direct back-projection (BP) in real space or Fourier reconstruction and SIRT (or SART), which compares reprojections of the reconstruction to the original dataset and iteratively minimizes the difference. Importantly, the contributions of the tilt views in reciprocal space represent a structure of co-axial tilted planes, so the sampling of spatial frequencies is non-uniform. BP and Fourier reconstruction, for example, result in over-weighting of the low spatial frequencies and a hazy appearance in virtual slices of the reconstructed volume. This is normally compensated by weighting to suppress the low and boost the high frequencies. The choice of weighting function strongly affects the texture of the image, and reconstruction becomes something of an art.

An inherent characteristic of BP is that a bright point in a projection forms a continuous ray in the reconstruction (see, for example, Fig. 6 in Carazo *et al.*, 2006 ▸). While the projections from multiple views converge to a spot at the point of intersection, the contrast between this intersection and the rays themselves is only on the order of the number of tilts. When viewed at a distant plane in the reconstruction, these rays will appear as a series of spots. Considering that all points in the projections, of any intensity, radiate to rays, the result is ‘salt and pepper’ noise that permeates the reconstructed volume. Unlike random noise due to low electron fluence or imperfect detectors, or the contribution of background noise from the embedding vitreous ice or carbon support (Baxter *et al.*, 2009 ▸), the origin of this structural noise is geometrical, arising from redistribution of the specimen contrast based on the geometry of the tilt scheme. Such a geometrical artifact may be described as a convolution with a kernel, and a means to calculate this kernel was proposed based on sampling theory (Seifer, 2023 ▸; Seifer *et al.*, 2024 ▸). Note that the reconstructed volume also involves a convolution with the point spread function (PSF) of the physical illumination by the electron beam. Since the kernel is itself a finite array of voxels, it is calculated by accurate integration over the intensity of the scanning beam. These two features are effectively combined into a single kernel by summation of multiple copies of the single beam representing the optical illumination, tilted to the set of angles used in generating the reconstruction. Due to its appearance, we call this kernel the ‘fanout’ PSF (Fig. 1 ▸).

In a number of earlier works, we have demonstrated that contrast in BP reconstructions is qualitatively improved by 3D deconvolution (Waugh *et al.*, 2020 ▸; Kirchweger *et al.*, 2023 ▸; Croxford *et al.*, 2021 ▸; Seifer *et al.*, 2024 ▸). The kernel represents the idealized BP reconstruction of a bright point under the physical illumination of a particular experimental condition. The most obvious results are noise reduction in 2D virtual sections and a very significant improvement in the axial, *i.e.* depth, resolution, resulting in greatly enhanced interpretability.

The most successful and stable algorithm in our experience has been the entropy-regularized, iterative deconvolution (ERDC) originally developed for fluorescence microscopy by Muthuvel Arigovindan at UC San Francisco (Arigovindan *et al.*, 2013 ▸). It is based on the use of a unique regularization term with intensity-dependent penalization of the second-order derivatives. This approach manages to suppress noise effectively while at the same time preserving essential details, and the processing is robust to noise even after many iterations. Unfortunately, the code is not open source nor actively maintained, and sustainability has become a major concern. Execution might also be accelerated and the accessible volume expanded by taking advantage of modern advances in computer architecture such as GPUs. Moreover, our established workflow combines several different software environments that more strongly reflect the history of the development than proper requirements for implementation. Therefore, we undertook a rewriting of the code from scratch, inspired by the original publication but geared more specifically to the tomography application. We expect that the resulting software presented here should be accessible and conveniently applicable by the community to 3D reconstruction originating from both STEM and TEM modalities.

## 2. Application

In order to demonstrate the application of 3*dcon*, we used a previously published dual-axis cryo-STET (data can be found on Zenodo at https://doi.org/10.5281/zenodo.14959968 and in the Electron Microscopy Data Bank as EMDB entry EMD-51764; Kirchweger, Wolf *et al.*, 2025 ▸) dataset of adherent mouse cells from our laboratory (Fig. 2 ▸) and a well known TEM-based cryo-ET (EMPIAR-10164; Schur *et al.*, 2016 ▸) dataset of HIV-1 virus-like particles (VLP; Fig. 3 ▸). Processing begins with a simple (unweighted) BP reconstruction, *i.e.* after the series alignment as might be performed in *IMOD* or *AreTomo* and, in the TEM case, after correction of the contrast transform function (CTF). A fanout PSF is then generated with a beam profile appropriate to the angles used in BP. (Note that these may differ from the precise goniometer angles if optimized in alignment, namely the .tlt versus the .rawtlt file in *IMOD*.) Deconvolution is then executed as an iterative procedure. In each case, we aimed for 3*dcon* settings that produce high-quality images at a small number of iterations (*e.g.* around 20) while remaining stable over a much larger number (200–500).

Dual-axis tomography reduces the large missing wedge to a missing cone, resulting in a similar appearance for *xz* and *yz* orthosections of the reconstruction. As such, it affords a good opportunity to focus on the denoising effect of deconvolution on the small missing-wedge artifacts. The data were acquired using a workflow that we described recently for microscopes equipped with a dual-axis stage (Kirchweger, Wolf *et al.*, 2025 ▸); the *SerialEM* script automatically returns to the same list of stage locations (Navigator points) after rotation of the grid by 90°. The reconstruction presented in Fig. 2 ▸ displays an unweighted BP, a SIRT-like filter and processing by 3*dcon* with 20 iterations. It is well known that the simple back-projection operation results in a relative down-weighting of the high spatial frequencies (Radermacher, 2006 ▸). Compensatory weighting with a ramp filter was a classical solution, but high-frequency noise is amplified together with the signal. A current favorite is the SIRT-like filter, or ‘fake SIRT’, which introduces a sculpted ramp function that empirically imitates the contrast produced by iterative SIRT reconstruction for a specified number of iterations, without the heavy penalty in processing time. The improved interpretability is immediately apparent (Fig. 2 ▸*b*). 3*dcon* brings a further improvement (Fig. 2 ▸*c*). In the *xy* view of the mitochondria, membranes are well separated and protein densities decorating the cristae are resolved in a sample 550 nm thick (Fig. 2 ▸*c*). The improvement afforded by 3*dcon* is most apparent in the *xz* and *yz* slices, where the image sharpness is significantly improved. Note that the dual-axis acquisition effectively symmetrizes these two orthogonal cross-sections. For comparison, conventional reconstructions from the first-acquired ‘*a*’-axis tomogram appear in Supplementary Fig. S1. There, the improvement in the less well represented *xz* section is even more dramatic.

3*dcon* processing involves a number of adjustable parameters to tune the regularization, as well as the overall number of iterations. Parameter choice and significance are described in Section 3. The smoothing parameter has the dominant effect, as anticipated. Examples appear in Supplementary Fig. S2. We additionally compare with the output of the original ERDC, with parameters chosen for visual similarity. While we have attempted to maintain consistency in notation with the original software, the numerical values are not identical and so must be evaluated anew. Iterative deconvolution methods may also be subject to divergence of noise amplification with an unlimited number of cycles (Sage *et al.*, 2017 ▸). In order to test this, we ran 3*dcon* for 200 iterations and observed no instability or hint of divergence at high spatial frequencies.

The current benchmarks for tomogram denoising are *cryoCARE*, based on the Noise2Noise model (Buchholz, Jordan *et al.*, 2019 ▸; Buchholz, Krull *et al.*, 2019 ▸), and *IsoNet*2 (Liu *et al.*, 2025 ▸), based on deep learning of a filter based on small subvolumes of the reconstruction. For comparison with 3*dcon*, we applied both methods to the *a*-axis dataset of the cell tomogram, first splitting the series into reconstructions generated from even and odd tilts. *CryoCARE* (Supplementary Fig. S3*b*) produces a visually pleasing result, albeit smoother than that of 3*dcon*. *IsoNet*2 produces very strong contrast at membranes, but areas within the mitochondrial matrix appear to be smeared (Supplementary Fig. S3*c*). Possibly this could be improved by fine-tuning internal parameters such as the box size for network training. The comparisons, shown in Supplementary Fig. S3, should therefore be regarded as indicative rather than conclusive.

To demonstrate that 3*dcon* can also be applied to TEM-based cryo-ET data, we chose a publicly available dataset of virus-like particles (VLP) from HIV-1 (EMPIAR-10164; Schur *et al.*, 2016 ▸). For tomogram generation, we followed the pipeline in *nextPYP* (Liu *et al.*, 2023 ▸). We generated a contrast transfer function (CTF)-corrected reconstruction by unweighted BP, then a fanout PSF using the refined tilt angles and finally a deconvolution using 3*dcon*. Fig. 3 ▸ shows the improvement of 3*dcon* starting from BP by comparing *xy* planes and orthoplanes of the tomogram. In the BP tomogram (Fig. 3 ▸*a*) the outline of the VLP is clearly visible, but details are hard to see. Weighted back-projection using the SIRT-like filter improves the contrast very substantially (Fig. 3 ▸*b*). However, 3*dcon* (Fig. 3 ▸*c*) more clearly recovers the CA-SP1 hexamer on the VLP. Again we explored the effects of smoothing parameters and a large number of iterations, with exemplary results shown in Supplementary Fig. S4. Lower smoothing values retained a higher level of detail, as expected, and no high-frequency divergence was observed even for 500 iterations. We also ran the two denoising routines described above. For the VLP dataset, both *cryoCARE* (Fig. 3 ▸*d*) and *IsoNet*2 (Fig. 3 ▸*e*) appear to be more effective than 3*dcon* in quieting the background. The particle density itself appears quite similar for *cryoCARE* and 3*dcon* processing. In terms of visual impression, *IsoNet*2 generates a cleaner image and performs a much better missing-wedge correction, as indicated by the generation of a round VLP viewed in all three orthogonal sections. Also, the white ‘wings’ surrounding the gold nanoparticles are most effectively suppressed. However, it tends to remove weaker densities, for example in the center of the VLP, and to suppress potentially unique features such as the four dark spots along the inner perimeter at the bottom of the VLP. Certain artifacts also appear in the particles themselves, most notably features of inverted (white) contrast within the protein shell. 3*dcon* should be robust against such features that might be introduced by methods based on deep learning.

Attempting to quantify the enhancements seen by eye, we took the approach of self-Fourier shell correlation (SFSC) as reported recently (Verbeke *et al.*, 2024 ▸). It is based on half-datasets selected from odd–even subsamplings from the reconstructions, evaluated per axis so as to avoid skipped pixels as in a checkerboard sampling. As shown in Supplementary Fig. S5, the unweighted BP performs poorly, with a significant improvement seen for the SIRT-like reconstruction. The evaluated performance of 3*dcon* and *IsoNet*2 is essentially identical, with *cryoCARE* yielding the most impressive curve at the highest spatial frequencies. Note that the curves do not decay to 0 because of the strong binning (by 6) imposed on the original dataset to facilitate processing of the entire tomogram.

Finally, we benchmarked the execution time of 3*dcon* in comparison with the original ERDC, as well as with *cryoCARE* and *IsoNet*2 on the same two datasets, as detailed in Table 1 ▸. For the comparison we used CPU and GPU server hardware, as well as an Apple MacBook Air for 3*dcon*. The execution time for 3*dcon* was very fast, on the order of 1 min for the VLP and 10 min for the cellular tomograms. This suggests its utility for routine incorporation into the initial processing workflow, which would enable the selection of optimal data for further evaluation using the heavier deep-learning methods.

## 3. Implementation

Before we begin with theoretical considerations, one practical pitfall should be kept in mind. The basic assumption of deconvolution is that the empty space is represented by zeros. Conventional TEM and STEM bright-field images, where the background is bright, can also be handled by 3*dcon* if the --negative flag is specified, or if the contrast of the tomogram is inverted beforehand. A dark-field-like contrast, on the other hand, allows visualization with dark contrast mapped to transparency so that bright objects appear suspended in space (Kirchweger, Seifer *et al.*, 2025 ▸).

Deconvolution aims to invert the effect of the convolution between an object *g*(**r**) and a kernel *h*(**r**) that occurs during data acquisition, resulting in the measured data *f*(**r**): 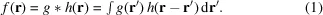


It is essential that the kernel depends only on the relative positions **r** − **r**′. Not all processes satisfy this condition. Important examples that can be approximated as convolutions include fixed out-of-focus purely geometric blurring, constant-velocity motion blur, parallel beam tomography followed by simple BP, and diffraction-limited imaging in the weak phase limit. Since a composition of multiple convolutions is also a convolution, a single deconvolution operation can in principle invert them all simultaneously.

Artifacts in data acquisition for STEM tomography arise from both the diffraction-limited probe and the BP reconstruction. Due to the finite convergence angle α of the focused illumination, the former can be approximated as a simple convolution for thin specimens,
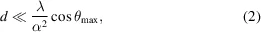
where *d* is the specimen thickness, θ_max_ is the maximum tilt angle and λ is the electron wavelength. Similarly, for TEM tomography, where the significant defocus Δ*f* required to generate adequate contrast is also responsible for the image blurring, the specimen thickness is also limited: 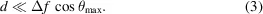


Deconvolution is traditionally formulated as a minimization problem, 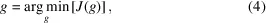
where *J*(*g*) is a loss function that quantifies the quality of the result *g*(**r**) given the input data and prior knowledge. The loss function suggested in Arigovindan *et al.* (2013 ▸) consists of three terms, 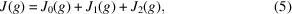
where the regularization *J*_1_ and positivity *J*_2_ terms impose prior knowledge about the object, while *J*_0_ enforces agreement with the input data. The latter is estimated as the sum of squares difference 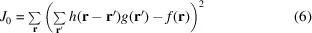
between the input data *f*(**r**) and the result convolved with the kernel *h*(**r**). This kernel, also known as the point spread function (PSF), in the case of STEM tomography reconstruction is the fan-shaped response to a point source after BP. For convenience, we built two separate tools: (i) *fancy*, to generate the PSF, and (ii) 3*dcon*, to actually perform the deconvolution. Alternatively, the deconvolution may be performed using an externally generated PSF.

### 3.1. PSF generation with *fancy*

The diffraction-limited probe in STEM is approximated by a parallel beam with an Airy disk intensity profile,
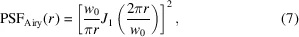
where *J*_1_ is the Bessel function of the first kind, *r* is the radial distance from the center of the probe and *w*_0_ is given by 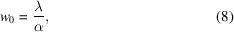
with λ being the electron wavelength and α the convergence semi-angle. The complete PSF is a superposition of such probes for all beam directions, *i.e.* for all tilt angles. Of course, STEM uses a focused illumination; the parallel beam condition (equation 2) expresses the presumption that the specimen is thinner than the depth of focus. Alternatively, if the depth-dependent magnification is taken into account, a 4D STEM image may be computationally refocused by techniques based on parallax (for example tcBF; Yu *et al.*, 2025 ▸), synchronization (shadow montage; Seifer *et al.*, 2026 ▸) or ptychography using a deliberately defocused probe (Varnavides *et al.*, 2026 ▸).

For an accurate deconvolution, the PSF must be represented as a 3D voxel array at least twice the size of the input volume in each direction. Each voxel is assigned the integrated PSF intensity, which presents major implementation difficulties because of the array size. This is partially alleviated by the fact that the beams are sparse, and contribution of the parts beyond a few units of *w*_0_ can be neglected. Nevertheless, an efficient integration algorithm is needed to compute the PSF in reasonable time (Fig. 4 ▸). This is achieved by the following protocol.

(i) Numerically pre-computing integrals of equation (7) on an adaptive fine 1D partition.
(ii) Generating a 2D polar grid using the partition from the previous step.
(iii) Assigning integrated profile values to each 2D grid cell.
(iv) Approximating full beam as a superposition of straight rays emitted from the centers of each 2D grid cell.
(v) Tracing each ray in space and summing their contributions to the 3D PSF voxel array.
(vi) Repeating the previous two steps for all beam directions.

This approach, as implemented in *fancy*, allows arbitrary beam orientations, such as those used in dual-axis tomography or single-particle analysis, and provides the flexibility to accommodate other beam profiles than the simple Airy disk.

### 3.2. Deconvolution with 3*dcon*

We largely follow the notation of Arigovindan *et al.* (2013 ▸), where the ERDC software is introduced. We emphasize here only the important implementation decisions that are not explicitly detailed in that work, or where our approach differs from the original paper.

ERDC stands out by its use of the entropy-based regularization term *J*_1_, which contains second spatial derivatives of the reconstruction ∂^2^*g*/∂*x*^*i*^∂*x*^*j*^ to penalize high spatial frequency components. One can build two quadratic scalars from these derivatives: the sum of squares of all second derivatives and the square of the Laplacian. We combine both scalars in our implementation: 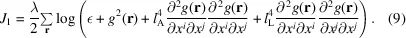


Characteristic lengths for the two types of smoothing *l*_A_ and *l*_L_ can be set independently and specified either in voxel dimensions or in angstroms. The parameter λ controls the overall strength of the regularization, while ε is adjusted to tune the contributions of intensity and smoothing terms. We use symmetric stencil finite differences to compute all derivatives in real space. For completeness, we also explicitly write down the positivity constraint term 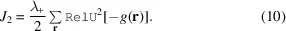


All code is implemented in Python, utilizing flexible CPU/GPU vectorized operation via PyTorch (Ansel *et al.*, 2024 ▸) wherever the computation speed is critical. Other parts rely on the widely used NumPy library (Harris *et al.*, 2020 ▸).

Depending on available hardware, the code allows parallel execution and features fast execution even on large volumes. This is exemplified in Table 1 ▸, where we compare ERDC (on a cluster node with 48 CPUs) with 3*dcon* (on a node with two GPUs, eight CPUs and 24 GiB RAM, and a MacBook Air with eight M2 CPUs and 24 GiB RAM).

Special care is taken to reduce memory consumption by optionally splitting the volume into chunks. Although the PSF itself is not separable, the Fourier transform is separable, allowing us to perform convolution sequentially. This approach also enables the utilization of GPUs with limited memory or effective use of the CPU cache. There is no hard upper limit to the tomogram size as had been the case for ERDC. However, 3*dcon* runs of tomograms that do not fit into local memory significantly prolong running times.

The loss function minimization is performed via a robust conjugate-gradient method. We used a ‘monkey-patched’ scipy.optimize.fmin_cg function from the SciPy library (Virtanen *et al.*, 2020 ▸) to reduce memory consumption by avoiding unnecessary copies of large arrays. The variations of all three terms of the loss function with respect to the reconstruction *g*(**r**) are computed analytically,
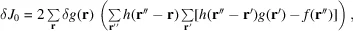

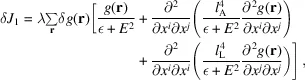
where *E*^2^ is the sum of the last three terms under the logarithm in equation (9) and 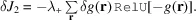


The package is open source for wide usage and flexible for extension to other regularization terms or use in other projects as a library. It is available at https://github.com/elbaum-lab/3dcon.

### 3.3. Tomogram alignment and reconstruction

The dual-axis cryo-STET dataset was aligned in *IMOD* (Kremer *et al.*, 1996 ▸; Mastronarde, 1997 ▸; Mastronarde & Held, 2017 ▸) as described previously (EMDB entry EMD-51764; Kirchweger, Wolf *et al.*, 2025 ▸).

The HIV-capsid dataset has been described previously (EMPIAR-10164; Schur *et al.*, 2016 ▸). The dataset was imported to *nextPYP* (Liu *et al.*, 2023 ▸) and aligned using fiducials.

For the HIV-capsid dataset, all tomograms were CTF-corrected as part of the *nextPYP* workflow. Tomograms from even and odd tilt series were produced for the *a* axis of the STEM and HIV-capsid dataset for input into *cryoCARE* and* IsoNet*2.

As a starting model for 3*dcon* we used unweighted back-projection tomograms. These were produced in *IMOD* by setting the flag RADIAL to 0.5 0.5. Alternatively, *Tomo*3*D* (Agulleiro & Fernandez, 2011 ▸, 2015 ▸) can be used when using the flag -w off. Additional SIRT-like (Zeng, 2012 ▸) filtered tomograms were produced in *IMOD*.

### 3.4. Fanout generation

The fanouts were generated using the bundled *fancy* program. The program takes refined tilt-series parameters (either as standard input or a .tlt file format from *IMOD*). It reads the size of the tomogram, if a reference tomogram is defined. Other important parameters are --alpha (the convergence angle α) and --wavelength (λ). For the STEM datasets, α and the λ were chosen as the settings from the microscope, while all the other parameters were kept as defaults.

For processing externally CTF-corrected TEM data sets, 3*dcon* is tasked with exclusively removing back-projection artifacts. This can be achieved by using a PSF composed of infinitely thin beams. To force *fancy* to ignore the Airy profile and to generate appropriate PSF we set the absolute rendering beam radius --rabs to a value smaller than the pixel size in angstroms.

The --crop option was activated in all cases, which cuts most of the empty volume of the PSF.

### 3.5. Deconvolution runs

To execute 3*dcon* runs, we set the two types of smoothing *l*_A_ and *l*_L_ to 1 pixel and adapted λ and ε.

3*dcon* settings in the single-axis STEM example shown here included λ set to 0.001 and ε to 0.0001 in Supplementary Figs. S1(*c*) and S1(*d*) (20 and 200 iterations, respectively). 3*dcon* settings in the dual-axis STEM example shown here included λ set to 0.001 and ε to 0.0001 (Fig. 2 ▸*c* and Supplementary Fig. S2*b*; 22 and 200 iterations, respectively), and λ set to 0.001 and ε to 0.0001 (Supplementary Figs. S2*c* and S2*d*); 21 and 200 iterations, respectively).

In the TEM example, settings included λ set to 0.001 and ε to 0.0001 (Figs. 3 ▸*g*–3 ▸*i* and Supplementary Fig. S4*a*; 23 and 500 iterations, respectively), and λ set to 0.01 and ε to 0.0001 (Supplementary Figs. S4*b* and S4*c*; 21 and 500 iterations, respectively). As a starting point, we suggest keeping ε at the default value and performing several runs with λ values of, for example, 0.1, 0.001 and 0.0001. In general, higher smoothing generates stronger contrast but less fine details, while lower smoothing retains the finer details at the expense of contrast. All tomograms were kept at bright-field contrast, and the --negative flag was set for 3*dcon* to take care of inverting the intensity.

### 3.6. Visualization

All tomograms were visualized in *ChimeraX* (Pettersen *et al.*, 2021 ▸), except for those in Supplementary Fig. S4 which were saved from 3*dmod* (Kremer *et al.*, 1996 ▸).

### 3.7. SFSC calculation

To calculate the SFSC, we followed the procedure proposed in Verbeke *et al.* (2024 ▸).

## 4. Discussion

3D deconvolution is an effective approach to ameliorate the reconstruction artifacts of BP from a discrete set of view angles (Waugh *et al.*, 2020 ▸; Kirchweger *et al.*, 2023 ▸; Seifer, 2023 ▸; Seifer *et al.*, 2024 ▸). While these artifacts are recognized as distortions that appear in orthoslices along the untilted view direction (*i.e.xy* or *xz*), they are also a primary cause of noise in virtual sections throughout the depth (*i.e.* cuts in *xy*). It should be emphasized that the relevant noise is structural and geometric, originating in the illumination pattern, in contrast to background scattering, for example from the vitreous ice, or statistical due to low electron count or poor detector efficiency. As such, the standard Poissonian or Gaussian noise models are suitable by coincidence at best. Deconvolution should in principle be an integral step in reconstruction by BP. To date, 3D deconvolution has been applied mainly to cryo-STEM tomography, where the artifacts are exaggerated by sample thickness (Waugh *et al.*, 2020 ▸; Kirchweger *et al.*, 2023 ▸; Kirchweger, Seifer *et al.*, 2025 ▸; Kirchweger, Wolf *et al.*, 2025 ▸; Elbaum, 2018 ▸; Elbaum *et al.*, 2021 ▸), and the ER (entropy-regularized) algorithm (Arigovindan *et al.*, 2013 ▸; Seifer *et al.*, 2024 ▸) was found to work most effectively. A similar approach was used for dual-axis tomography, resulting in near-isotropic resolution across the orthoslices (Kirchweger *et al.*, 2023 ▸). Use of ER deconvolution has also been demonstrated for wide-field TEM tomography (Croxford *et al.*, 2021 ▸). Recently, a similar approach was employed for molecular data processed by single-particle analysis. There, it was shown that deconvolution reduces anisotropy in the reconstruction that originates from preferred orientations (Li *et al.*, 2025 ▸). We should emphasize that ERDC is a distinct operation from that of contrast transfer function (CTF) correction or low-frequency contrast enhancement as offered in *Warp* (Tegunov & Cramer, 2019 ▸), both of which rely on a similar mathematical operation but implement differently and use a different kernel.

The goal of this work has been to adapt the original ERDC software for electron tomography and to modernize it in light of subsequent developments in computer hardware. 3*dcon* is written in Python and distributed as open-source software under the GPLv3 license for sustainability. While maintaining the original features, several specializations and extensions have been added. In particular, the package includes a second regularization option based on the square of the Laplacian and a convenient PSF generator *fancy* for tilt tomography. The latter appears as a fanout rather than a radially symmetric cone as in fluorescence microscopy. The code can take advantage of thin, nearly planar fanouts to reduce memory requirements and accelerate execution, but it is also ready to work with space-filling kernels (for example in dual-axis configurations.)

The algorithm was specifically optimized for memory efficiency, allowing efficient CPU cache usage and GPU utilization when available. By taking advantage of modern Python libraries, the code is flexible with respect to hardware, running on multiple CPUs and up to two GPUs. Although a server is recommended, 3*dcon* runs even on a laptop computer. A very practical aspect of the improved running speed is the ability to rapidly explore the parameter space of the deconvolution for a given specimen type. This process frequently requires multiple attempts and can significantly improve the final result.

As one of the benefits of 3*dcon* is denoising, we compared the results with the widely used SIRT-like filter (Zeng, 2012 ▸), as implemented in *IMOD* (Kremer *et al.*, 1996 ▸; Mastronarde & Held, 2017 ▸), *cryoCARE* (Buchholz, Jordan *et al.*, 2019 ▸; Buchholz, Krull *et al.*, 2019 ▸) and *IsoNet*2 (Liu *et al.*, 2025 ▸), popular tools in the TEM-based cryo-ET workflow. When comparing with the simple SIRT-like filter for frequency-weighting the back-projection, 3*dcon* shows higher contrast by reducing the salt-and-pepper noise in the *xy*, *yz* and *xz* planes, revealing the CA-SP1 hexamers even in the *yz* view. *CryoCARE*, on the other hand, uses a neural net to compare half-datasets in order to isolate and suppress noise on the assumption of statistical independence between the halves. *IsoNet*2 also uses half-datasets and employs a subvolume-averaging approach to learn about the noise in the tomogram. The underlying architecture of *Isonet* and its very impressive performance in improving axial resolution for the VLPs suggest that the U-Net filter generated from numerous subtomograms might represent a 3D PSF. The axial resolution in the case of the thicker mitochondrial tomogram is less impressive, however. The training to recognize the missing wedge and develop a suitable filter must come from redundancies within the dataset itself. This might explain the variable performance in these two examples. In STEM tomography we do not acquire multiple movie frames per tilt image, so two tomograms were reconstructed from the even and odd tilts. The resulting noise reduction by both *cryoCARE* and *IsoNet* was impressive, especially considering that the underlying noise model is not fully justified. However, we found that the 3*dcon* processing better preserves the finer detail. Moreover, it takes a purely algorithmic approach that does not depend on training generated from the recorded data *per se*. Therefore it is likely to work even when neural networks may not. Provided that the PSF is an accurate description of the acquisition conditions (*i.e.* correct alignment, beam aberrations, absence of nonlinear effects) 3*dcon* should provide a precise regularization for a given set of adjustable weights and iterations. Due to the fast execution speed, we propose that 3*dcon* should be incorporated in the standard reconstruction pipeline, where it could serve for immediate visualization as well as a screening tool for the more time- and resource-consuming deep-learning methods. We expect that it will become a useful instrument in the tool-chest for electron tomography.

## Supplementary Material

Supplementary Information. DOI: 10.1107/S205979832600999X/sor5008sup1.pdf

## Figures and Tables

**Figure 1:**
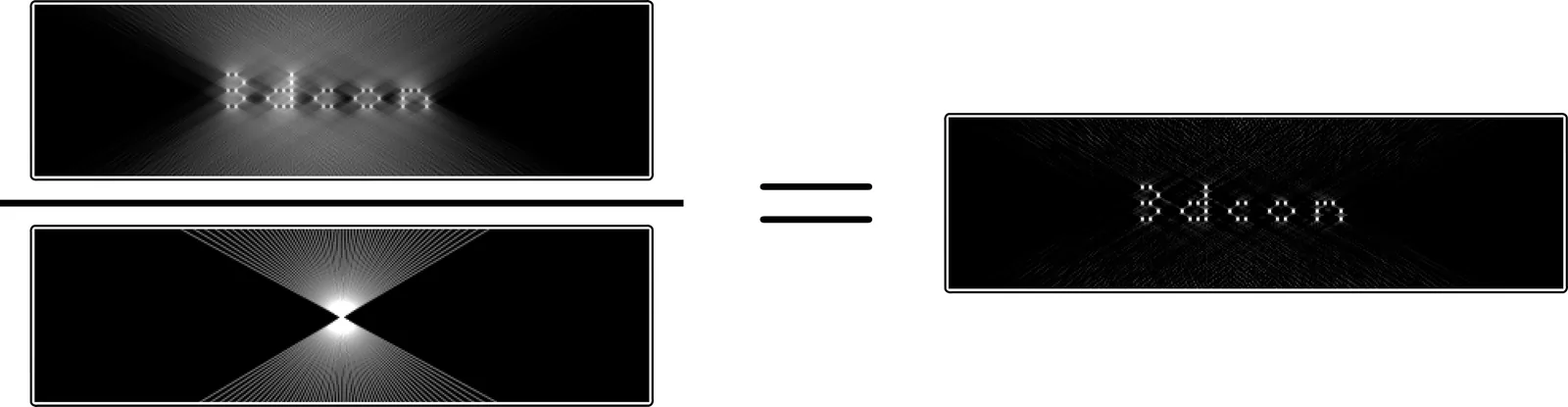
Simulation of 3*dcon* processing with a phantom of isolated particles. A tomographic reconstruction suffers characteristic artifacts related to missing wedges, large and small. These are seen most strikingly in a cross-section perpendicular to the tilt axis. Deconvolution with a point spread function (PSF, shown in denominator) representing the tilt geometry effectively suppresses these artifacts.

**Figure 2:**
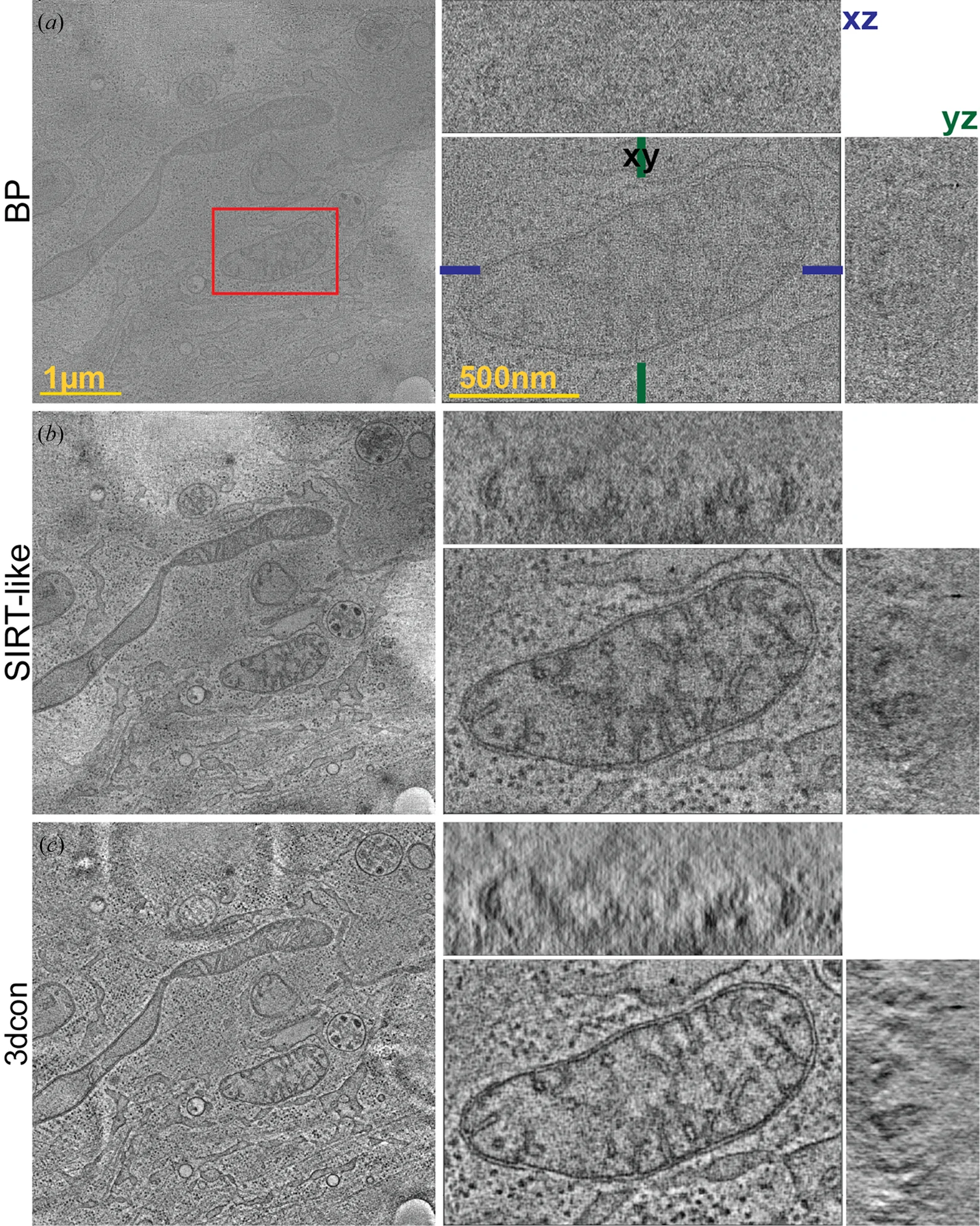
Comparison of (*a*) BP, (*b*) SIRT-like and (*c*) 3*dcon* for a dual-axis cryo-STET dataset (EMDB entry EMD-51764; Kirchweger, Wolf *et al.*, 2025 ▸). The first column shows individual slices of the whole tomogram (scale 1). The second column zooms into one mitochondrion, as highlighted by the red box (scale 500) and shows the *xy*, *xz* and *yz* slices. Blue and green lines in the *xy* plane indicate the positions of the *xz* and *yz* planes.

**Figure 3:**
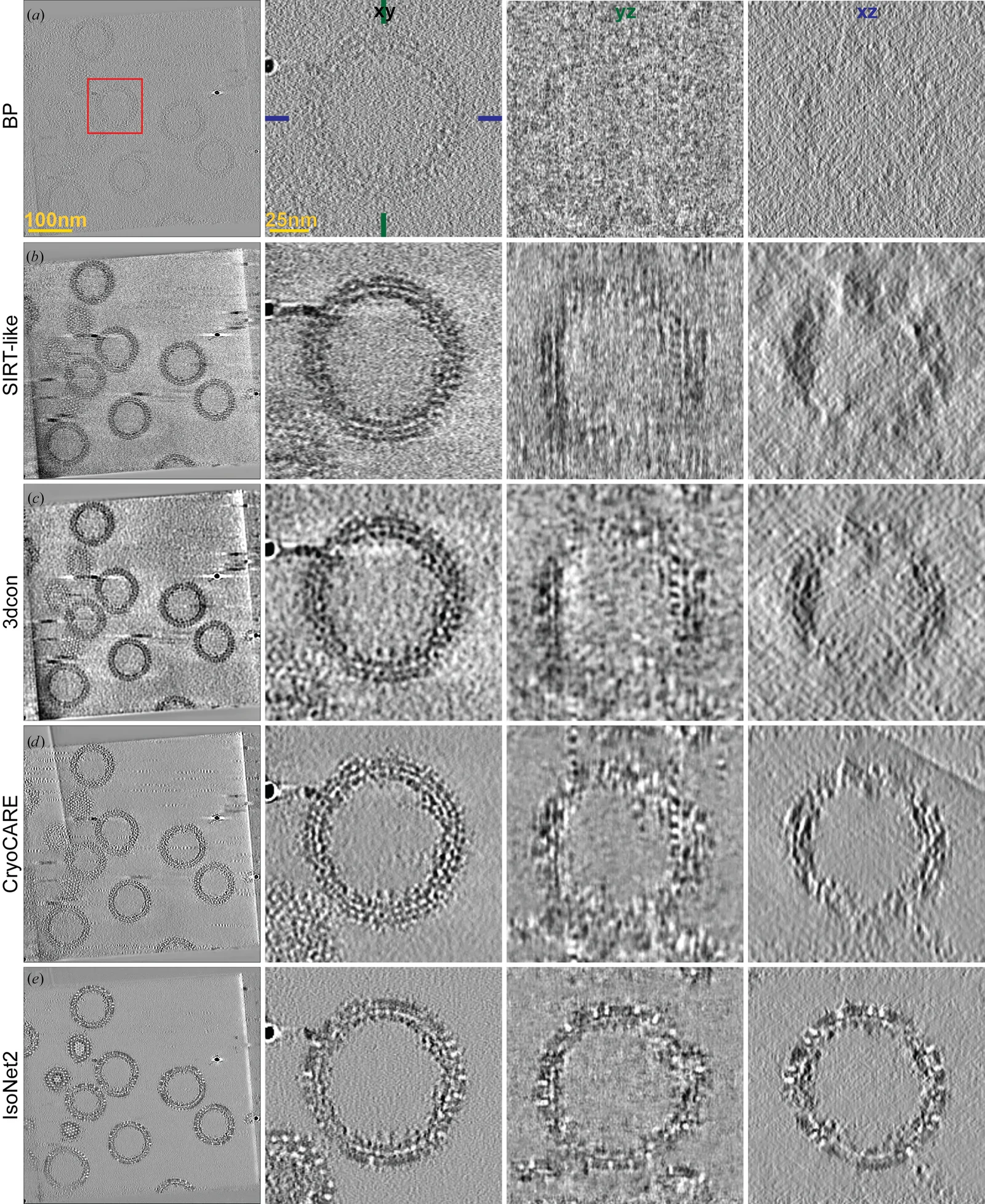
A cryo-TEM tomogram (EMPIAR-10164; Schur *et al.*, 2016 ▸) processed with 3*dcon*: (*a*) the unweighted BP, (*b*) the SIRT-like filtered BP, (*c*) the 3*dcon*, (*d*) *CryoCARE* and (*e*) *IsoNet*2 of a slice in the tomogram. The first column shows the *xy* virtual section of the entire field of view; scale 100). The second, third and last columns show the *xy*, *xz* and *yz* cross-sections of a VLP (scale 25) highlighted by the red box. Blue and green lines in the *xy* plane indicate the positions of the *xz* and *yz* planes.

**Figure 4:**
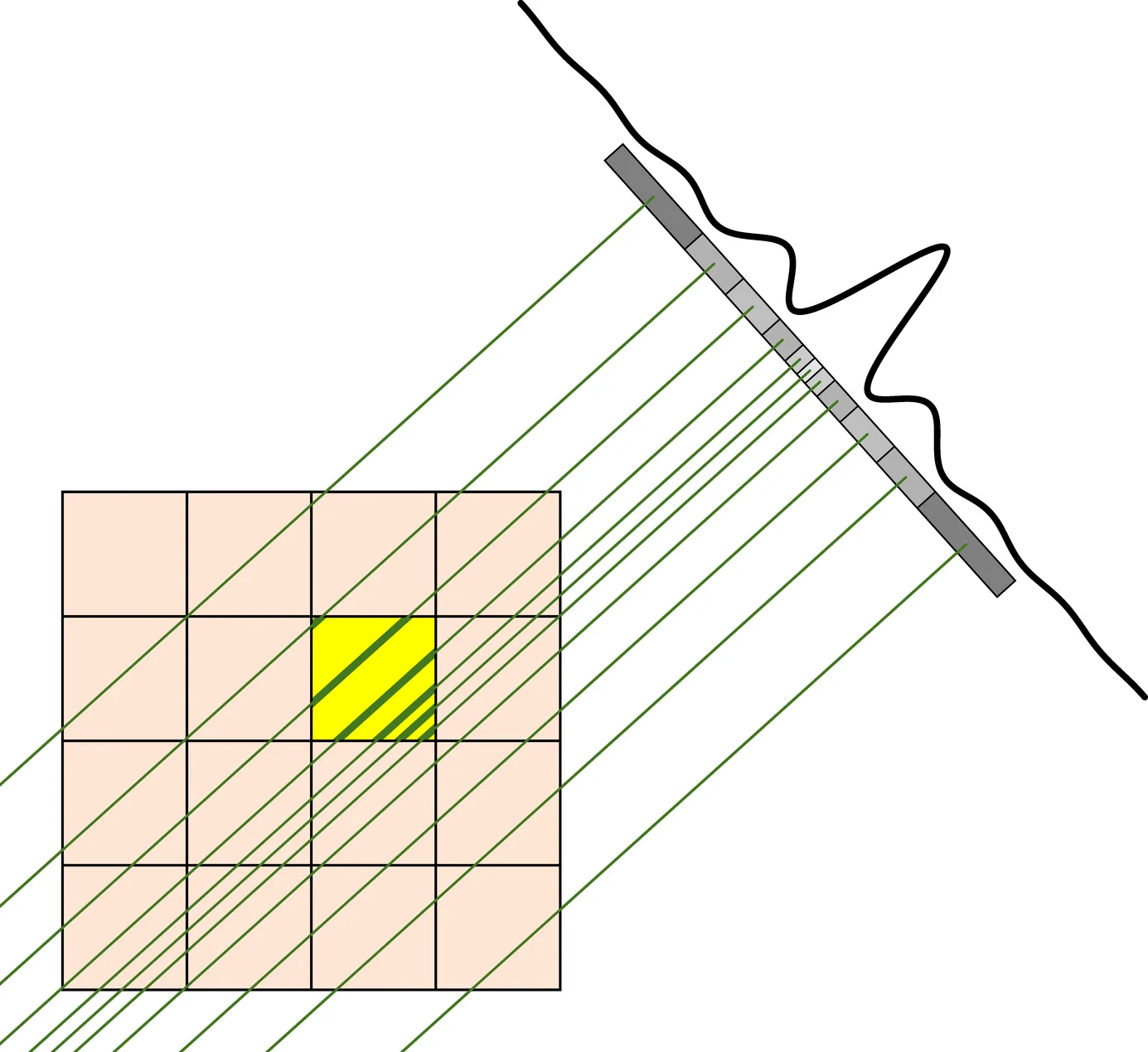
Schematic representation of the PSF rendering algorithm. The contributions of each ray for all beam directions are summed to produce the final PSF voxel array.

**Table 1:** Execution times

Program	Hardware	Process	Processing time (min)
Tomogram size: 2048 × 2048 × 238 pixels (Fig. 2 ▸)			
ERDC	48 CPUs, 256 GiB RAM	Fanout generation	75
		Core2decon	89
3*dcon* (20 iterations)	2 NVIDIA L40S GPUs, 24 CPU cores, 70 GiB RAM	Fanout generation	2
		3*dcon*	8
*CryoCARE*	2 NVIDIA L40S GPUs, 32 CPU cores, 128 GiB RAM		43
*IsoNet*2	4 NVIDIA L40S) GPUs, 40 CPU cores, 128 GiB RAM		230
Tomogram size: 648 × 648 × 341 pixels (Fig. 3 ▸)			
ERDC	48 CPUs, 256 GiB RAM	Fanout generation	5
		Core2decon	8
3*dcon* (20 iterations)	2 NVIDIA L40S GPUs 8 CPU cores 24 GiB RAM	Fanout generation	0.2
		3*dcon*	1
	Apple MacBook AirM2: 8 CPU cores, 24 GiB RAM	Fanout generation	0.2
		3*dcon*	17
*CryoCARE*	2 NVIDIA L40S GPUs, 32 CPU cores, 64 GiB RAM		38
*IsoNet*2	2 NVIDIA L40S GPUs, 32 CPU cores, 64 GiB RAM		190 (÷5)

## Data Availability

The software described in this article is available from the Elbaum Lab GitHub page https://github.com/elbaum-lab/3dcon and is distributed as open source under a GPLv3 license. The example datasets cited in the manuscript are available through Zenodo, EMDB and EMPIAR as indicated in the text.
