# Supplementary material for: 3dcon: tomogram denoising by deconvolution
Source: Acta Crystallogr D Struct Biol. 2026 Oct 12;82(Pt 11):1240–50. doi: 10.1107/S205979832600999X (PMC13717825; doi:10.1107/S205979832600999X)
Supplement: Supplementary file 1 [file d-82-01240-sup1.pdf]

Supplementary Material: 3dcon: tomogram denoising by deconvolution

Peter Kirchweger[† a], Lev Melnikovsky[† a], Shahar Seifer[a], and Michael Elbaum[a]

[a]Department of Chemical and Biological Physics, Weizmann Institute of Science, 7610001 Rehovot, Israel

[†]These authors contributed equally

1 Supplementary Figures

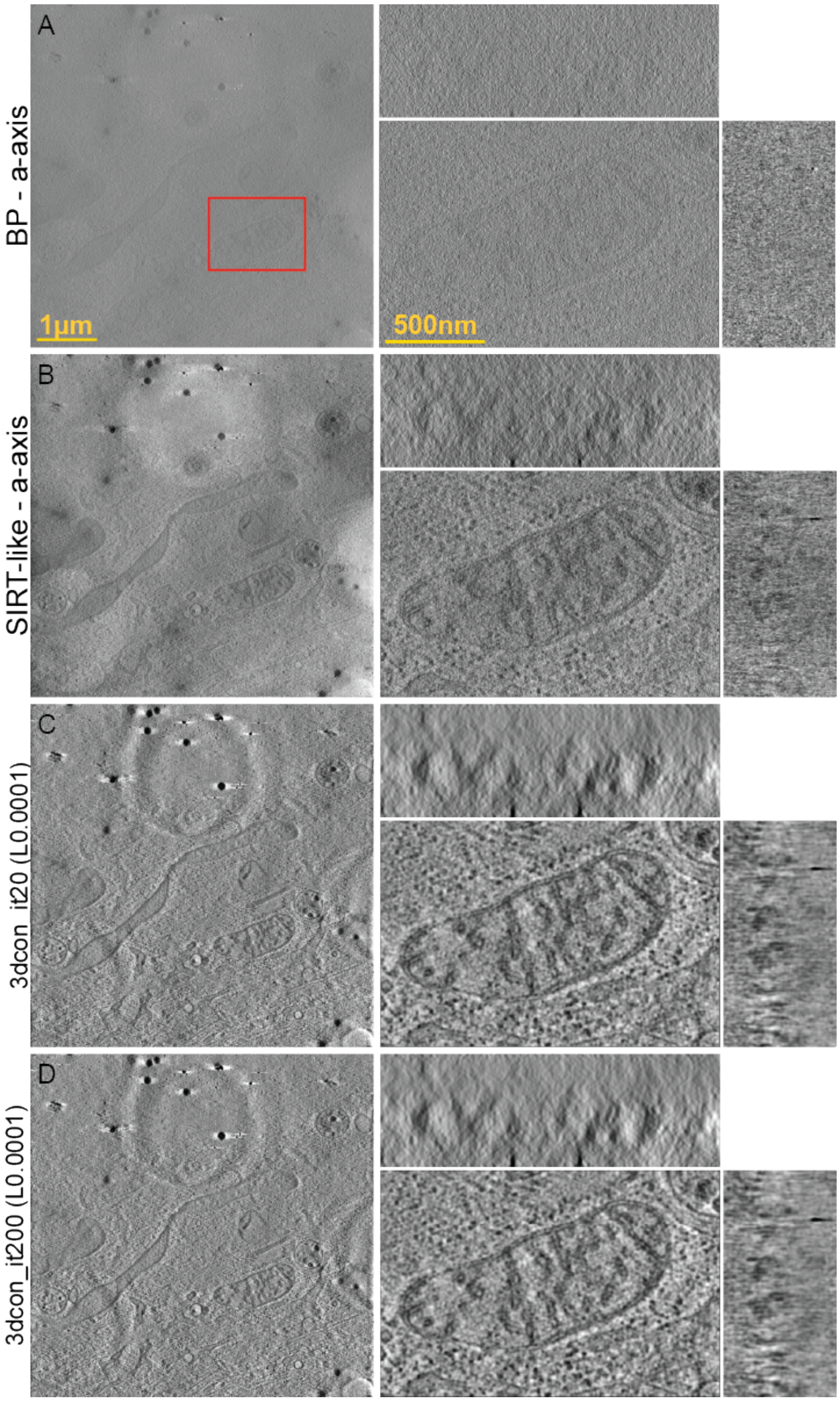

Figure S1: Comparison of (A) BP, (B) SIRT-like, `3dcon` (C) 20 iterations, and (D) (200 iterations)) of the a-axis cryo-STET dataset from (EMD-51764, Kirchweger *et al.*, 2025). 1st column show individual slices of the whole tomogram (scale: 1 µm). 2nd column zooms into one mitochondrion, as highlighted by the red box (scale: 500 nm), and shows the *xy*, *xz* and *yz* slices.

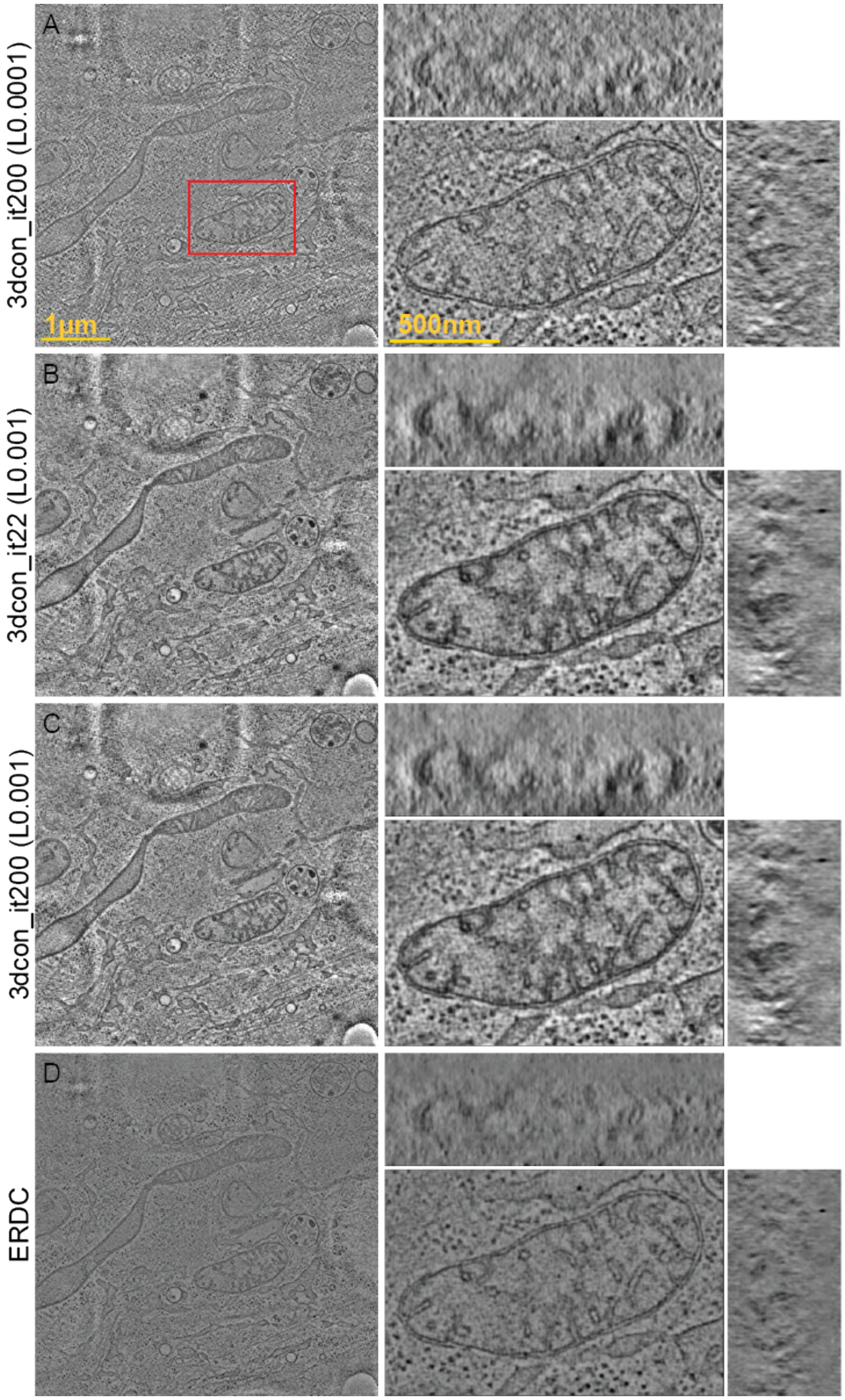

Figure S2: Comparison of different iterations and smoothing factors using `3dcon` (first three rows) and ERDC (bottom row) of the dual-axis cryo-STET dataset (EMD-51764, Kirchweger *et al.*, 2025). 1st column show individual slices of the whole tomogram (scale: 1 µm). 2nd column zooms into one mitochondrion, as highlighted by the red box (scale: 500 nm), and shows the *xy*, *xz* and *yz* slices.

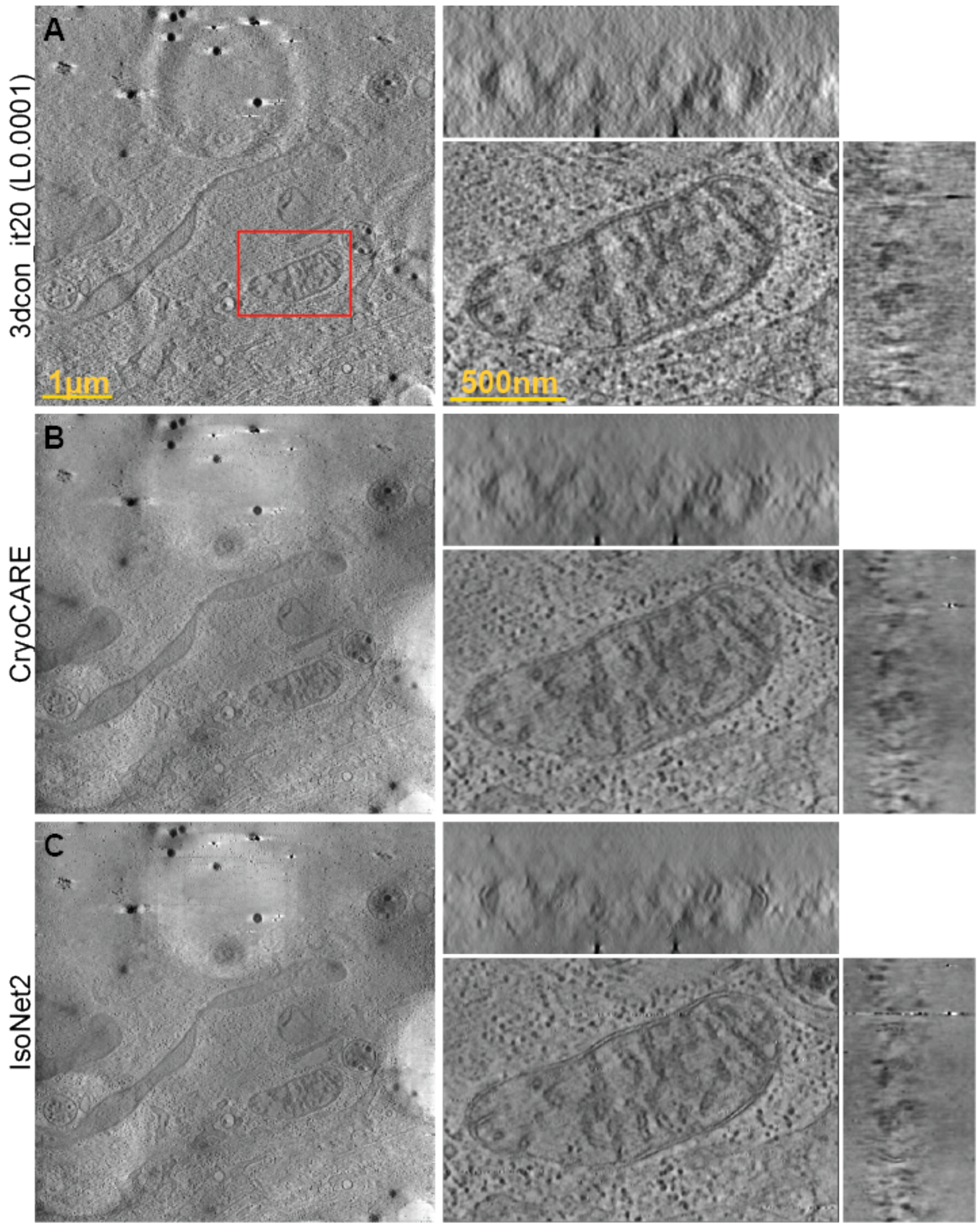

Figure S3: Comparison of (A) `3dcon` to (B) CryoCARE and (C) IsoNet2 of the a-axis of the cell tomogram. 1st column in all rows shows individual slices of the whole tomogram (scale: 1 µm (A-B) and 100 nm (C-E)). 2nd column zooms into one mitochondria, as highlighted by the red box (scale: 500 nm), and shows the xy, xz and yz slices. Panel (A) displays the same images as in Fig 2C.

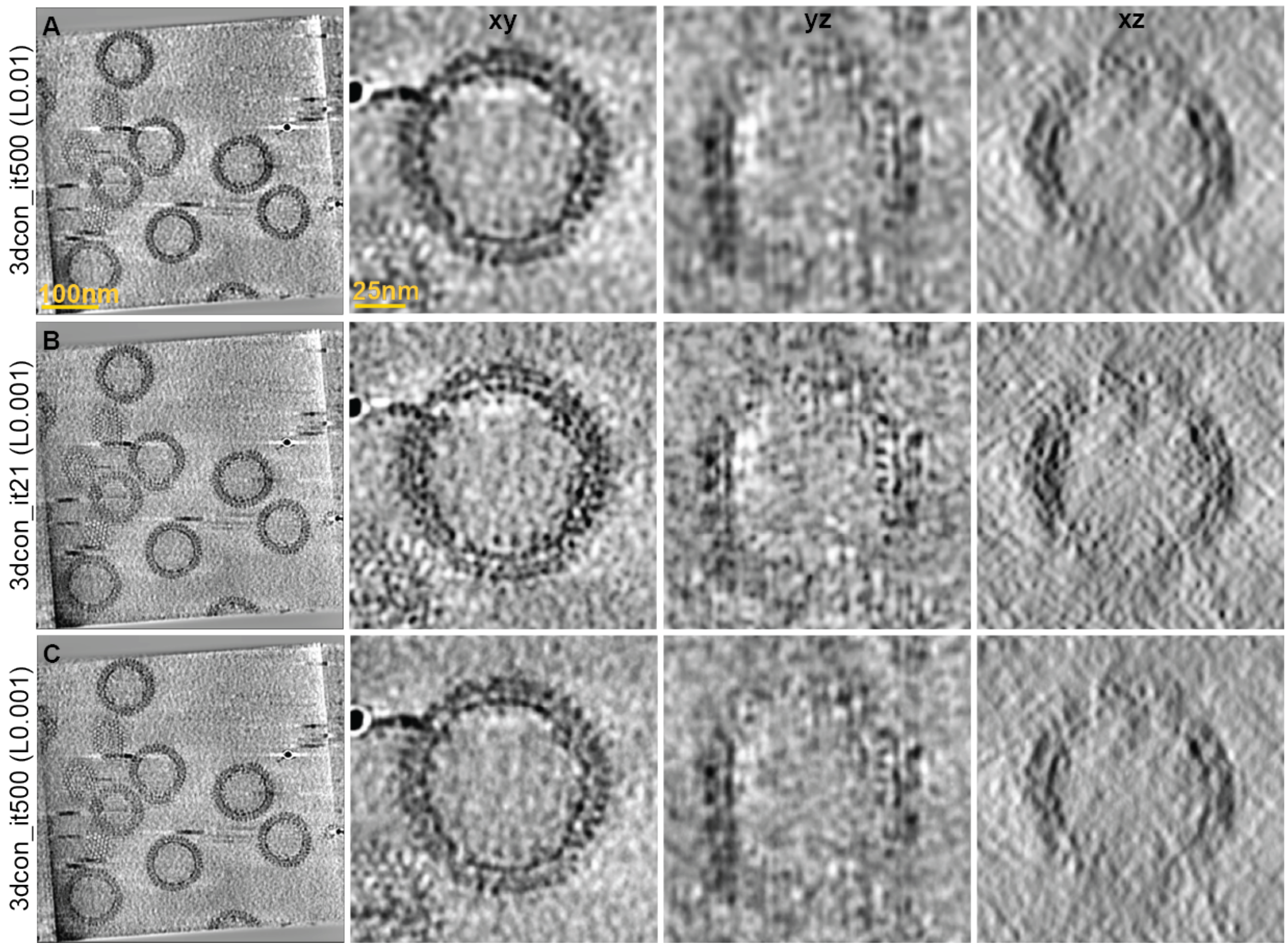

Figure S4: Comparison of different iterations and smoothing factors of a `3dcon` (A-C) of the cryo-TEM dataset (EMPIAR-10164, Schur *et al.*, 2016). The first column shows the xy of the whole field of view, scale: 100 nm). The second, third and last column show the xy, xz and yz of a VLP (scale: 25 nm) highlighted by the red box in Fig 3A of the main text.

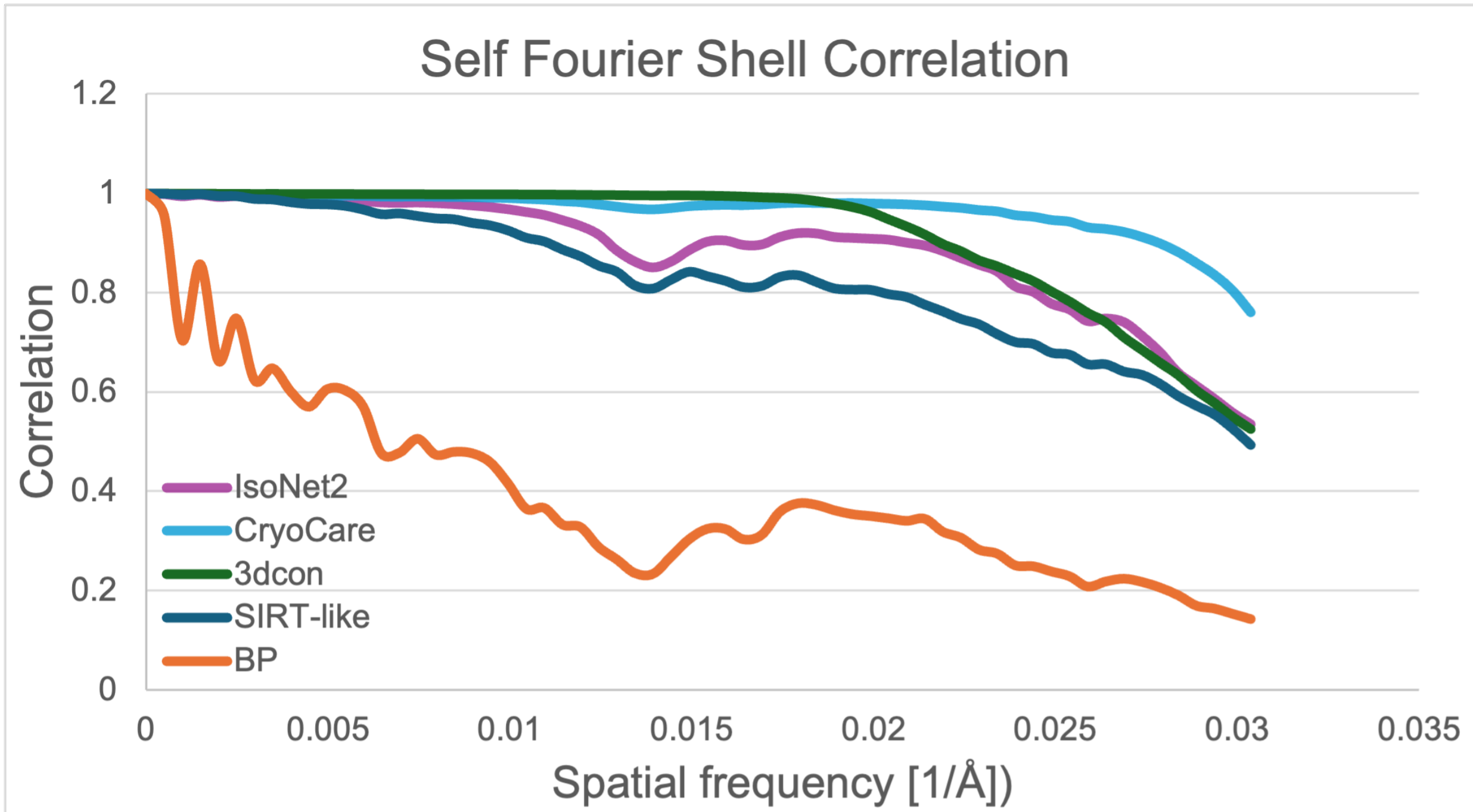

Figure S5: sFSC (Verbeke *et al.*, 2024) of the BP (orange), SIRT-like (blue), `3dcon` (green), CryoCARE (cyan) and IsoNet2 (purple) of the VLPs.

References

Kirchweger, P., Wolf, S. G., Varsano, N., Dadosh, T., Resch, G. P. & Elbaum, M. (2025). *Journal of cell science*, **138**(9), jcs263639.

Schur, F. K. M., Obr, M., Hagen, W. J. H., Wan, W., Jakobi, A. J., Kirkpatrick, J. M., Sachse, C., Kräusslich, H.-G. & Briggs, J. A. G. (2016). *Science (New York, N.Y.)*, **353**(6298), 506–508.

Verbeke, E. J., Gilles, M. A., Bendory, T. & Singer, A. (2024). *Communications Biology*, **7**(1), 101.
